# Flexible Pressure Sensor With Multi‐Stage Microdome Structure Enabling Ultra‐Wide Linear Range and High Sensitivity for Wearable Applications

**DOI:** 10.1002/advs.74927

**Published:** 2026-03-20

**Authors:** De‐Yu Kong, Kai Wang, Jia‐Nan Ma, Bo‐Ma, Teng‐Teng Gao, Hai‐Wang Zhang, Hong‐Tao Wang, Qiang Li, Hui Gao, Sheng‐Bo Sang

**Affiliations:** ^1^ Shanxi Key Laboratory of Artificial Intelligence & Micro Nano Sensors College of Integrated Circuits Taiyuan University of Technology Taiyuan China; ^2^ School of Materials Science and Engineering Tianjin University Tianjin China

**Keywords:** high sensitivity, multi‐stage microdome, MXene, pressure sensor, ultrawide linear range

## Abstract

Flexible piezoresistive sensors show significant application potential in wearable health monitoring, motion recognition, and human–machine interaction. However, current sensors often face a trade‐off between insufficient sensitivity and rapid saturation at high pressures, making it difficult to simultaneously achieve both high sensitivity and a wide pressure detection range. Herein, we propose an MXene‐based flexible piezoresistive sensor with a multi‐stage microdome (MSM) structure, enabling high sensitivity and a broad response range. Benefitting from the multi‐stage compression and stepwise contact feature of this architecture, the prepared MXene‐based flexible pressure sensor achieves an ultra‐wide detection range of up to 2500 kPa and a high sensitivity of 11.57 kPa^−^^1^. Coupled with deep learning algorithms, the designed palm‐ and sole‐shaped sensor arrays enable effective Morse code recognition, object grasping recognition, and human motion recognition, achieving a high recognition accuracy exceeding 95%. The developed MSM architecture presents a promising strategy for resolving the longstanding challenge of balancing high sensitivity and extended pressure detection range in piezoresistive sensors, offering an effective pathway to promote their large‐scale implementation.

## Graphical abstract

An MXene‐based flexible piezoresistive sensor featuring a multi‐stage microdome architecture is developed, which simultaneously realizes high sensitivity of 11.57 kPa^−^^1^ and an ultra‐broad detection range up to 2500 kPa. When integrated into palm and sole sensor arrays combined with deep learning algorithms, it achieves a recognition accuracy of over 95% in Morse code identification, grasping behavior analysis, and motion recognition.

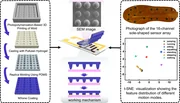

## 1 Introduction

As devices that convert external pressure into electrical signals, flexible pressure sensors [1, 2, 3, 4, 5, 6, 7] are critical to enabling tactile perception in human health monitoring [8, 9, 10, 11], motion detection [12, 13], intelligent robots [14, 15, 16], and human–machine interaction [17, 18, 19]. In recent years, with the advancement of wearable devices toward practicality, high precision, and long‐term battery endurance, developing flexible pressure sensors that synergize a wide linear range and high sensitivity has become a prominent research focus and key technical bottleneck in flexible electronics [20, 21]. High sensitivity enables accurate detection of subtle pressure changes, whereas a broad linear response ensures that sensor outputs maintain a direct proportionality to applied pressure [22, 23, 24], simplifying signal analysis and eliminating the need for complex data processing.

Among diverse pressure sensing mechanisms, piezoresistive pressure sensors have garnered significant attention owing to their simple structural design, facile fabrication process, stable output signals, and superior compatibility with flexible circuits. Typically, piezoresistive sensors function by transducing mechanical stimuli into measurable resistance changes, stemming primarily from adjustments to internal conductive networks, interfacial contact resistance variations, or microstructural deformation. Recent progress has focused on exploring diverse sensing materials to improve the sensing performance of flexible piezoresistive sensors. A range of conductive fillers such as carbon black [25, 26], graphene [27, 28, 29, 30], carbon nanotubes (CNTs) [31, 32], metal nanoparticles [33, 34], and MXenes [35, 36, 37, 38, 39, 40, 41, 42] have been embedded in elastomer matrices to produce composite materials for sensor applications. Commonly, these polymer‐filler composite architectures are often unable to meet the dual requirements of a broad detection range and high sensitivity. For these polymer‐filler composites, sensitivity is primarily determined by elastic modulus. Higher modulus enables broader detection ranges but sacrifices sensitivity [43, 44], while lower modulus improves sensitivity yet restricts the ability to measure large stresses [45, 46, 47]. This thus calls for composites that can undergo significant deformation across different ranges, in turn enabling marked variations in resistance.

To resolve this challenge, an effective method is designing hybrid microstructures with continuously varying contact states under varying pressure conditions, aiming to achieve an optimal balance between sensitivity and linear sensing range. For example, Zhang et al., [48]. designed a Janus conductive structure assembled with dual‐resistive sensitive layers for piezoresistive sensors, thereby attaining an ultra‐wide linear detection range (up to 3800 kPa) with a moderate sensitivity of ≈4 kPa^−1^. Zhou et al., [49]. developed a high‐performance flexible pressure sensor featuring a sensitivity of 24.6 kPa^−^^1^ and an ultra‐wide linear range of 1400 kPa, wherein pyramidal carbon foam arrays serve as sensing layers and the elastic spacer functions as a stiffness regulator. Taking inspiration from the highly sensitive microstructures of human skin, Wan et al., [50]. developed MXene‐decorated urchin‐like microstructures for the fabrication of flexible electronic sensors, which exhibited a high sensitivity of 784.02 kPa^−^^1^ but only within a pressure range limited to ≈20 kPa. In another work, Zhou et al., [51]. constructed a pressure sensor with an ultrahigh sensitivity of 924.4 kPa^−^^1^ using a double conductive layer with porous and interlocking structures. Nevertheless, this superior performance is restricted to a narrow pressure range up to 70 kPa. Even though these studies have enhanced sensor sensitivity or extended the linear sensing range to a certain extent, inherent contradictions persist unresolved: high sensitivity is generally limited to low‐to‐moderate pressures, while a wider linear range often sacrifices sensitivity. Consequently, designing flexible piezoresistive pressure sensors that can simultaneously deliver high sensitivity and an ultra‐broad linear detection range constitutes a core challenge.

In this work, we developed an MXene‐based flexible piezoresistive sensor incorporating multi‐stage microdome (MSM) structures for the improvement of sensitivity and linear detection range. The core of this design is the integration of multi‐stage microdome structures, where each primary and secondary microdome is decorated with twelve micro‐hemisphere array units on its surface. During pressure loading, the multi‐stage microdome structures can achieve multi‐stage compression and stepwise contact, notably alleviating signal saturation issues in conventional microstructures. Resultant piezoresistive sensors exhibit an ultra‐wide pressure detection range of up to 2500 kPa, achieving a high sensitivity of up to 11.57 kPa^−1^ within 111–2500 kPa and excellent linearity (linear correlation coefficient R^2^≈0.998). Furthermore, assisted by deep learning algorithms, we developed palm‐ and sole‐shaped sensor arrays and subsequently classified the acquired spatiotemporal pressure data, thus demonstrating the practical application potential of the device in intelligent health monitoring and human–machine interaction scenarios.

## 2 Experimental Section

### 2.1 Preparation of MXene Solution

To begin with, 2 g of LiF was slowly incorporated into 25 mL of concentrated hydrochloric acid (36–38 wt%), followed by continuous stirring for 30 min to ensure uniform dispersion. Over a duration of 10 min, the MAX phase (Ti_3_AlC_2_, purchased from Jilin 11 Technology Co., Ltd.) was then gradually added to the above‐mentioned solution. Subsequently, the reaction temperature was adjusted to 35°C, and continuous stirring was maintained for 48 h to complete the etching reaction. The resulting solution was diluted with deionized water, and high‐speed centrifugation (12 000 rpm) was performed to wash the product. This washing‐centrifugation cycle was repeated several times until the pH of the solution reached 5–6. The obtained sediment was sonicated in ethanol for 1 h, after which deionized water was added to the precipitate. The mixture was then sonicated for 30 min and centrifuged at 3500 rpm for 3 min. Through repeated collection of the upper dispersion phase, an MXene solution with a concentration of 4 mg mL^−1^ was successfully obtained.

### 2.2 Fabrication of the Pressure Sensor

A photocurable resin was used as the positive mold material, and the desired multi‐stage microdome structure template was fabricated using 3D printing. Pullulan was used as the negative mold material (the ratio of deionized water to pullulan is 1:0.25). The pullulan hydrogel was coated uniformly on the positive mold, and a vacuum dryer was used to remove trapped air. The sample was then air‐dried for 24 h to solidify. After peeling, the pullulan negative mold was obtained. We thoroughly mixed the PDMS base and curing agent at a 10:1 weight ratio and then poured 0.5 mL of the mixture onto each negative mold, ensuring uniform coverage, degassed using a vacuum dryer, and cured at 60°C for 1 h. Peeling off the PDMS film yielded a multi‐stage microdome‐structured PDMS layer, which was treated with oxygen plasma for 5 min to enhance hydrophilicity. The pre‐prepared MXene solution was drop‐cast onto the PDMS film at a coating density of 0.2 mg cm^−2^, followed by drying at 50°C for 1 h. The MXene–PDMS film was cut into squares (5 mm × 5 mm) and placed onto interdigitated electrodes on a polyimide substrate. Finally, the pressure sensor was encapsulated with polyimide tape to prevent moisture interference from the environment, yielding the final pressure sensor.

### 2.3 Material Characterization

Scanning electron microscopy (SEM) images and energy‐dispersive X‐ray spectroscopy (EDS) analyses were obtained using a JSM‐IT700HR scanning electron microscope. X‐ray diffraction (XRD) patterns were recorded on a Malvern Panalytical Aeris diffractometer equipped with Cu Kα radiation (λ = 1.5418 Å) over a range of 5–90°. Atomic force microscopy (AFM, Bruker Dimension Icon) and transmission electron microscopy (TEM, JEOL F200) were employed to characterize the microstructure, morphology and thickness of MXene sheets.

### 2.4 Pressure Performance Characterization

A pressure testing machine (Zhiqu, ZQ‐990B) was employed to apply external loads to the pressure sensor, while a digital multimeter (Keithley, DMM6500) was used for current measurement. All tests were conducted under ambient conditions of approximately 20°C and 30% relative humidity (RH).

To evaluate the sensing performance of the pressure sensor, we defined sensitivity as follows:

(1) $$S=\frac{\Delta I/I_{0}}{\Delta P}$$

where *I*_0_ represents the initial current in the unloaded state, Δ*I* denotes the current variation caused by external pressure, and Δ*P* represents the change in pressure.

## 3 Results and Discussion

### 3.1 Preparation and Characterization of the MSM‐Based Piezoresistive Pressure Sensor

**FIGURE 1:**
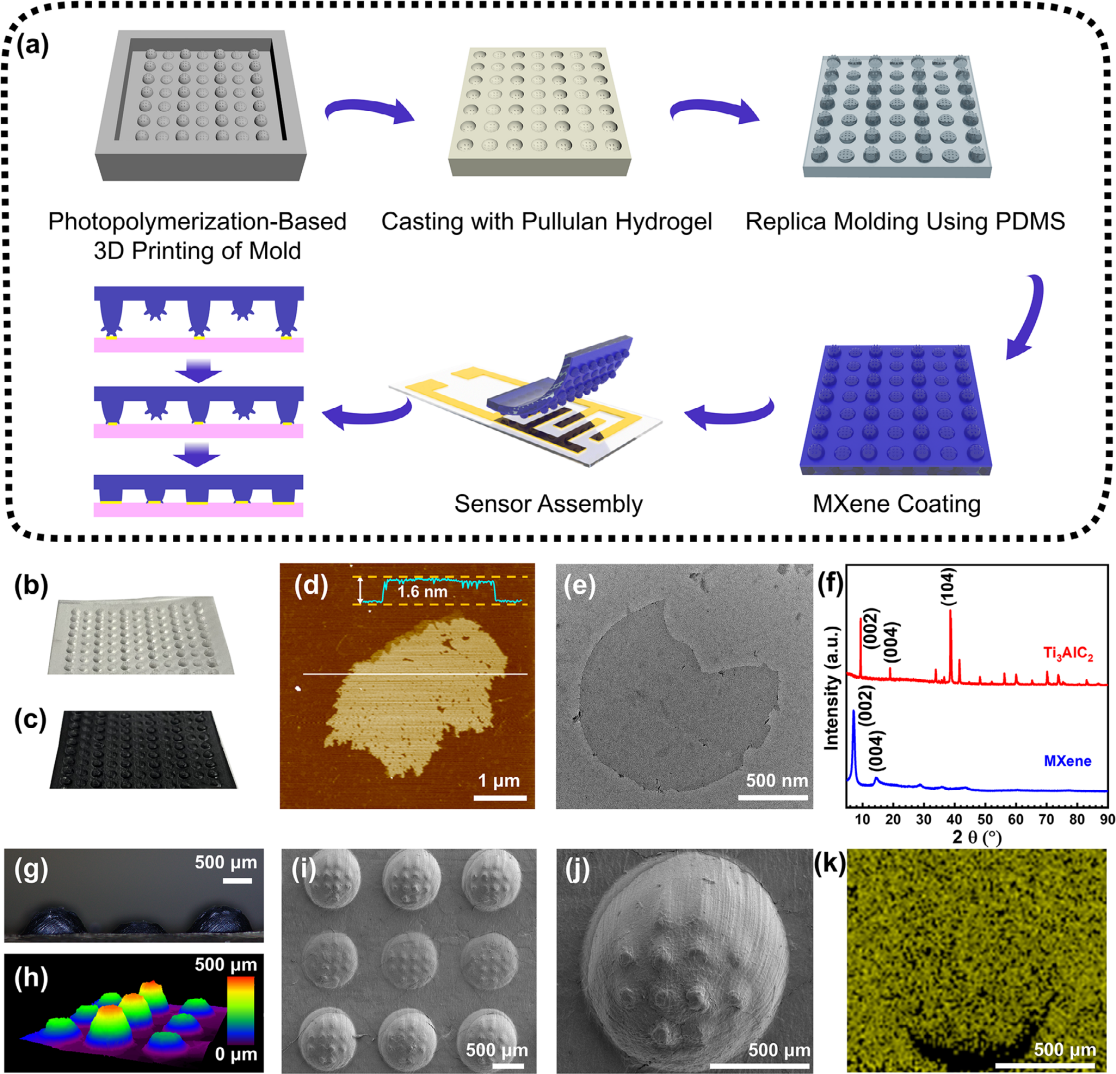
Fabrication process and structural characterization of the MSM‐structured pressure sensor. (a) Schematic illustration of the fabrication process of the MSM‐structured flexible pressure sensor. (b) Photograph of the MSM‐structured PDMS film without MXene drop‐coating. (c) Photograph of the MSM‐structured PDMS film after MXene drop‐coating. (d) AFM image of MXene flakes. (e) TEM image of MXene nanosheet. (f) XRD patterns of Ti_3_AlC_2_ and MXene. (g) Side‐view microscopic photograph of the MSM structure with a primary/secondary microdome height ratio of 1:0.5. (h) 3D optical microscopic scanning image of the MSM structure with a primary/secondary microdome height ratio of 1:0.5. (i) SEM image of the overall sensor. (j) Enlarged SEM image of a single microdome. (k) Ti elemental distribution map in the EDS mapping of the single microdome.

Figure 1a schematically depicts the fabrication process of the flexible MSM‐based pressure sensor, which is fabricated via a two‐step molding approach. First, a positive mold was prepared from a resin material using photocurable 3D printing. Subsequently, pullulan hydrogel was uniformly coated onto the positive mold; after drying and peeling, the negative mold was obtained. Finally, PDMS was cast onto the negative mold, and following curing and peeling, the MSM structures was transferred onto the PDMS film (Figure 1b). Casting MXene onto the PDMS film results in the formation of the pressure‐sensing layer (Figure 1c). By assembling this layer with interdigitated electrodes, the MSM‐based pressure sensor is successfully fabricated. In general, the performance of a sensor can be directly affected by its sensing material, hence we performed characterization on the MXene used in this study. As shown in Figure 1d, the AFM image reveals that the thickness of Ti_3_C_2_T_x_ MXene nanosheet is about 1.6 nm. The TEM image in Figure 1e shows that the single‐layer MXene exhibits a regular and well‐defined morphology. Additionally, compared with the XRD pattern of pristine Ti_3_AlC_2_ MAX, the (104) peak disappeared and the (002) peak exhibited a shift from 9.38° to 7.16° in the as‐synthesized MXene, which verifies the successful synthesis of MXene (Figure 1f). Furthermore, the XPS survey spectrum confirms the presence of Ti, C, and O elements in the as‐prepared MXene (Figure S1). The high‐resolution C1s spectrum can be deconvoluted into characteristic peaks attributed to C–Ti, C–C, and C–O bonds (Figure S2), which further verifies the successful removal of the Al layer and the formation of Ti_3_C_2_T_x_ MXene. The above results collectively demonstrate that the prepared MXene possesses high purity, which benefits the sensing performance of the sensor.

To visually demonstrate the MSM structure of the sensor, we conducted a series of morphological characterizations. The side‐view optical micrograph (Figure 1g) shows that with a primary/secondary microdome height ratio of 1:0.5, the primary microdome has a height of 500 µm, the secondary microdome 250 µm, and the spacing between adjacent structures is 500 µm. The 3D optical microscopic scanning image (Figure 1h) further confirms the morphology and structural parameters of the MSM structures. In addition, the 3D images of the positive mold, negative mold, and PDMS film with MSM structures before MXene coating (Figure S3) show that the two‐step molding process does not impair structural precision. To demonstrate the MSM structures more clearly, SEM images was provided. As presented in Figure 1i, twelve micro‐hemisphere array units are distributed on the surface of each primary and secondary microdome (diameter = 1 mm). Moreover, the magnified SEM micrograph of an individual primary microdome (Figure 1j) illustrates micro‐hemisphere structures of 100 µm diameter and 100 µm interspacing. Compared with the MSM‐structured PDMS film (uncoated with MXene, Figure S4), the EDS mapping of the MSM‐structured PDMS with MXene coating (Figure 1k) shows uniform Ti distribution, verifying homogeneous MXene coverage on the PDMS layer.

### 3.2 Sensing Mechanism and Performance of MSM‐Based Piezoresistive Pressure Sensor

**FIGURE 2:**
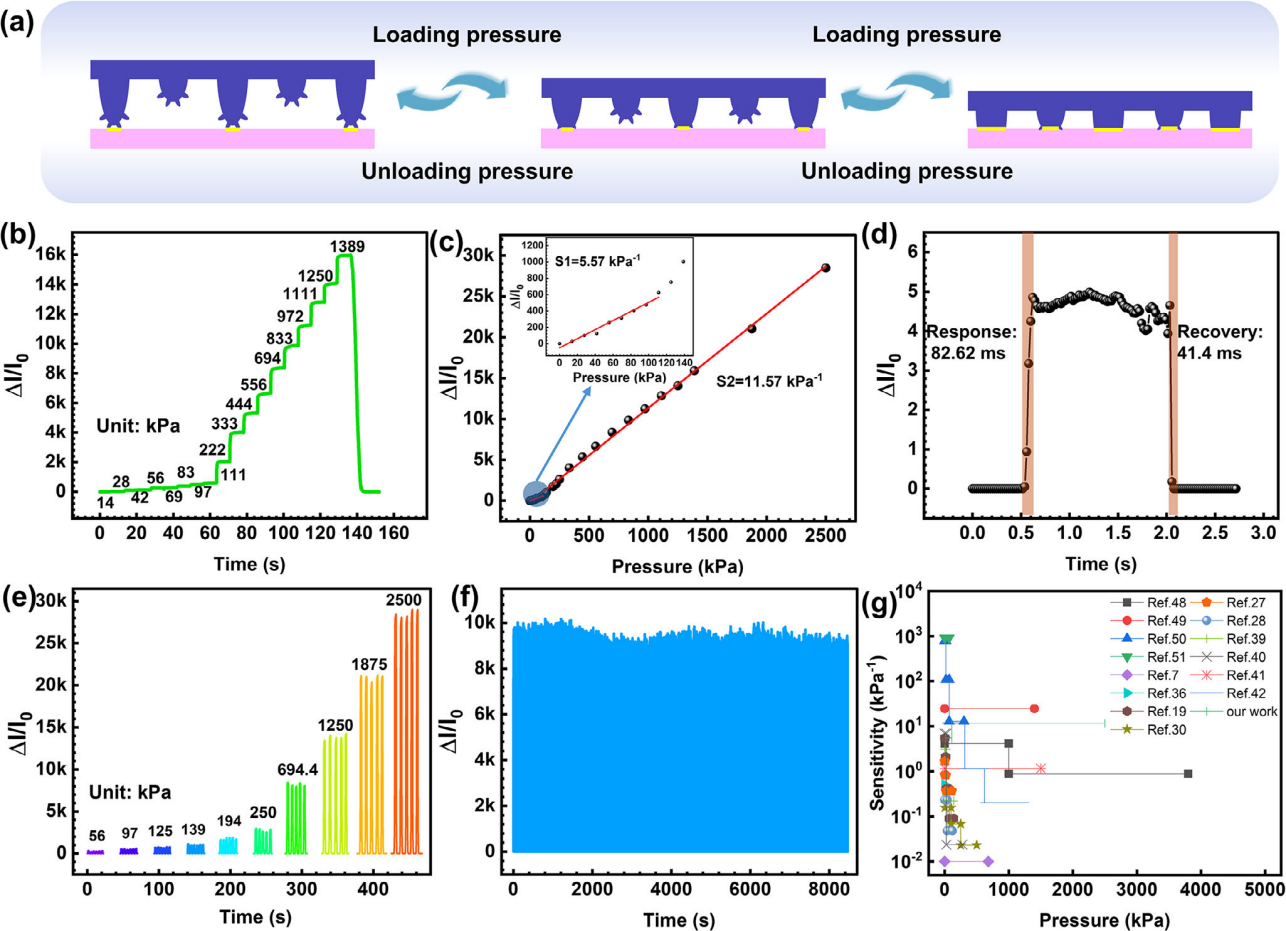
Sensing mechanism and performance characterization of the MSM‐structured pressure sensor. (a) Schematic diagram of the working mechanism of the MSM‐structured pressure sensor. (b) Δ*I*/*I*_0_‐pressure curve of the sensor under pressures ranging from 14 to 1389 kPa. (c) The sensitivity of the sensor under the external pressures. (d) Response and recovery time curves of the sensor under 1.4 kPa. (e) Repeatability output waveforms of the sensor under different pressures. (f) Durability test results of the sensor under 833 kPa (approximately 2900 pressure cycles). (g) Comparison of the sensitivity and range between MSM‐based sensor and previously reported sensors.

The sensing mechanism of the MSM‐based pressure sensor is illustrated in Figure 2a. Without external pressure, the micro‐hemisphere array of the sensing layer exhibits nearly point‐like contact with the electrode, generating high interfacial contact resistance that can effectively facilitate the improvement of the sensor's sensitivity. When external pressure is applied, the micro‐hemisphere array on the surface of the primary microdome structure is first compressed, increasing the contact area between the sensing layer and the electrode. Simultaneously, the stacked MXene nanosheets on the surface also undergo compression, reducing the interlayer spacing and effectively decreasing the thickness of the conductive layer. This compaction shortens the electron transport paths, increases conductive contacts between the nanosheets, and lowers the overall resistance, leading to an increase in the output current [52, 53, 54]. As the applied pressure increases, the primary microdome undergoes further compression. After the primary microdome is compressed beyond a certain threshold, the secondary microdome begins to make contact with the electrodes and provides additional conductive pathways, thus extending the sensor's pressure detection range. After releasing the applied pressure, the conduction pathways between the sensing layer and the electrode layer are disconnected.

To investigate the resistance response of the sensor, a simplified equivalent circuit diagram is presented in Figure S5. Briefly, the total resistance (*R*_total_) mainly consists of three major components: the resistance of the primary microdome (*R*_H_), the resistance of the secondary microdome (*R*_L_), and the resistance of the substrate (*R*_B_). It is worth noting that the resistance of the micro‐hemisphere array is included in the primary/secondary microdome for unified calculation. The resistance of each primary/secondary microdome includes the sidewall resistance (*R*_S_) and the contact resistance with the interdigital electrodes (*R*_C_). Under initial state, only the primary microdome structure with micro‐hemisphere array contacts the interdigital electrodes. Accordingly, the total resistance (*R*_total_) is primarily composed of *R*_H_ and *R*_B_, where *R*_H_ can be further divided into the initial contact resistance between the primary microdome and the electrode (*R*_HC_) and the initial sidewall resistance of the primary microdome (*R*_HS_). The corresponding formula is expressed as follows:

(2) $$R_{\mathrm{total}1}=R_{H}+R_{B}=R_{\mathrm{HC}}+R_{\mathrm{HS}}+R_{B}$$

Expressing the aforementioned equations in terms of the MXene’ resistivity (*ρ*), the radius of the primary microdome structure (*r*_H_), the initial contact area between the primary microdome structure and the electrodes (*A*_H_), the height of the primary microdome structure (*H*), and the thickness of the MXene layer (*T*_H_), the total resistance (*R*_total1_) can be expressed as:

(3) $$R_{\mathrm{total}1}=\frac{\rho T_{H}}{A_{H}}+\frac{\rho H}{T_{H}\cdot 2\pi r_{H}}+R_{B}$$

When the sensor undergoes low pressure, the primary microdome is compressed and deformed firstly, and the total resistance (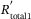) becomes:

(4)

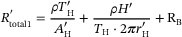




The resistance variation (Δ*R*_1_) can be expressed as:

(5)

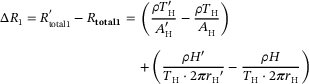




As pressure increases further, the secondary microdome starts to contact the electrodes and undergo compression, and the total resistance (*R*_total2_) is given by:

(6) $$R_{\mathrm{total}2}=R_{H}^{\prime }\left|\right|R_{L}+R_{B}$$

Similarly, by employing the resistivity of MXene (*ρ*), the contact radius (*r*_L_), the initial contact area (*A*_L_) between the secondary microdome structure and the electrode, the height (*L*) of the secondary microdome structure, and the MXene thickness (*T*_L_), the equation for the moment when the secondary microdome first contacts the electrode is given by:

(7)

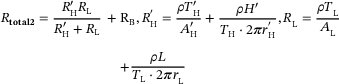




With further pressure loading, both the primary and secondary microdome structure undergo deformation, and the total resistance ($R_{\mathrm{total}2}^{\prime }$) can be written as:

(8)

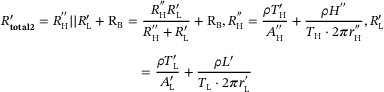




The resistance change (Δ*R*_2_) can be expressed as:

(9)

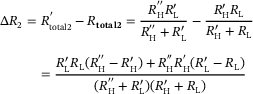




The sensor sensitivity can be expressed as $S=\frac{\Delta I/I_{0}}{\Delta P}=\frac{-\Delta R/R}{\Delta P}$, where Δ*R* denotes the change in resistance when applied pressure to the sensor, and *R* is the real‐time resistance under applied pressure. According to the above equations, the sensor sensitivity mainly depends on the initial contact area between the sensing layer and the electrode, the height ratio of the primary microdome to the secondary microdome, and the MXene thickness. For investigating these influencing factors, we fabricated sensors with diverse structures, including MSM structures with four different primary‐to‐secondary microdome height ratios (1:0.3, 1:0.5, 1:0.7, 1:0.9), single‐stage structures of varying heights (250 µm and 500 µm), and two‐stage structures. The MXene surface concentration was fixed at 0.15 mg cm^−^^2^. Figure S6 shows the side‐view microscopic images of sensors with different height ratios and surface morphologies. Following this, the sensors’ performance was characterized, and Figure S7a,b depicts the corresponding current‐pressure response curves and scatter plots. Additionally, Table S1 summarizes the segmented fitting data, which reveals that the MSM‐structured sensor with a primary‐to‐secondary microdome height ratio of 1:0.5 exhibits outstanding segmented linearity and high sensitivity. This phenomenon can be attributed to the existence of micro‐hemisphere array and the suitable height ratio of primary‐to‐secondary microdome. Thus, we selected this microdome configuration for further testing. Additionally, we investigated the effect of MXene thickness (adjusted by varying the MXene drop‐casting concentration) on sensing performance. Figure S8 illustrates the corresponding response curves and scatter plots, and Table S2 summarizes the segmented fitting results. The results demonstrate that the sensor featuring an MXene surface concentration of 0.2 mg cm^−^^2^ exhibits the optimal sensitivity and linearity among all tested samples. In summary, we selected the sensor with MSM structure (the primary‐to‐secondary microdome height ratio is 1:0.5) and an MXene surface concentration of 0.2 mg cm^−^^2^ for subsequent performance evaluation and application studies.

Figure 2b presents the current response of the sensor under stepwise pressure loading. With increasing pressure, the current change increases in a corresponding manner and maintains stability at each constant pressure level, indicating that the sensor exhibits good sensitivity and stability. To further evaluate the sensor's detection capability, we expanded the pressure loading range. As illustrated in Figure 2c, the sensor exhibits an ultra‐wide detection range of up to 2500 kPa. Specifically, in the low‐pressure regime (<111 kPa), it achieves a sensitivity of 5.57 kPa^−^^1^ with a correlation coefficient (*R* ^2^) of 0.973, while in the high‐pressure regime (111–2500 kPa), the sensitivity elevates to 11.57 kPa^−^^1^ with an *R* ^2^ value of 0.998. Such an enhancement arises from the secondary microdomes contacting the electrodes under increased pressure, which offers extra conductive pathways, significantly reduces the resistance, and thus improves the sensitivity in the high‐pressure region. As shown in Figure 2d, the pressure sensor exhibits a response time of 82.62 ms and a recovery time of 41.4 ms. Such rapid response to pressure variations enables its use in applications requiring immediate feedback, such as human–machine interaction and humanoid robotics. To examine the repetitive response under different pressure conditions, we conducted cyclic tests at various pressure levels. Figure 2e demonstrates that the sensor maintains outstanding repeatability and stability across all tested pressures. To meet the mechanical durability requirements for wearable devices, we further assessed its long‐term operational stability. At a pressure of 833 kPa, the sensor underwent approximately 2900 loading cycles (Figure 2f) with no noticeable performance attenuation, confirming its superior durability and repeatability. As illustrated in Figure 2g, a comparative analysis is conducted between our proposed MSM‐based pressure sensor and recently reported microstructured biomimetic pressure sensors. It is evident that our device exhibits superior performance in sensitivity and linear detection range. In addition, as shown in Figure S9, after being stored under different humidity conditions for three days, the sensor exhibits no observable performance degradation, indicating that the encapsulation effectively mitigates the influence of ambient humidity on sensing performance. Furthermore, Figure S10 presents the sensing performance measured under different pressures at 10‐day intervals over a 40‐day period. The sensor exhibits excellent long‐term stability with negligible variation. Overall, the multi‐stage microdome architecture provides a viable strategy for developing high‐performance sensors.

### 3.3 Wearable Applications Assisted by Machine Learning

High‐performance tactile perception is essential for human–machine interaction, wearables, and health monitoring. However, current wearable/skin‐attachable sensors suffer from limited sensitivity, narrow detection ranges, and insufficient spatial resolution, unable to meet the needs of fine tactile discrimination and complex dynamic analysis [55, 56]. The sensor proposed in this study exhibits high sensitivity, an extended operating range, robust stability, and customizable functionality, thereby meeting the diverse demands of practical applications. Moreover, when combined with machine learning, the arrays composed of these sensors can accomplish tactile sequence encoding and decoding, precise tactile object recognition, and gait pattern analysis. The data formats and training details related to the deep learning models in this study are provided in the Supporting Information.

**FIGURE 3:**
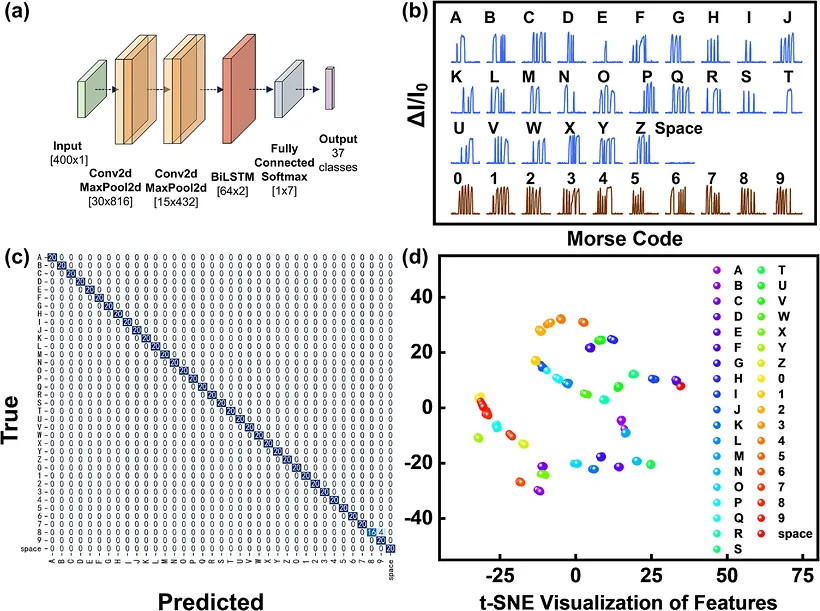
Morse code recognition enabled by the MSM‐structured sensor. (a) Schematic diagram of the CNN–BiLSTM model architecture. (b) Schematic diagram of Morse‐code pressing signals collected by a single sensor (“‐” and “.” distinguished by press duration). (c) Confusion matrix after 50 training epochs (test accuracy: 99.5%). (d) t‐SNE dimensionality‐reduction visualization showing the feature distribution of different classes.

To evaluate the dynamic signal–processing capability of the constructed sensor, we first employed Morse code recognition as a demonstration. A volunteer pressed the single sensor at a predefined rhythm, generating time‐series signals with distinct temporal and amplitude differences. We adopted the CNN‐BiLSTM‐based classification model illustrated in Figure 3a and performed 50 training epochs, thereby accomplishing effective Morse code decoding. Figure 3b illustrates representative input signals, demonstrating the high‐resolution output of the sensor. The confusion matrix in Figure 3c confirms strong consistency between the model predictions and the true labels, achieving an overall recognition accuracy of 99.5%. Additionally, t‐SNE visualization of the high‐dimensional features extracted by the convolutional neural network (CNN) in Figure 3d reveals that all 37 signal categories form 37 distinct clusters in the low‐dimensional space. This indicates that the model effectively learns and discriminates between different signal features, verifying both the reliability of the classification model and the stability of the sensor output.

**FIGURE 4:**
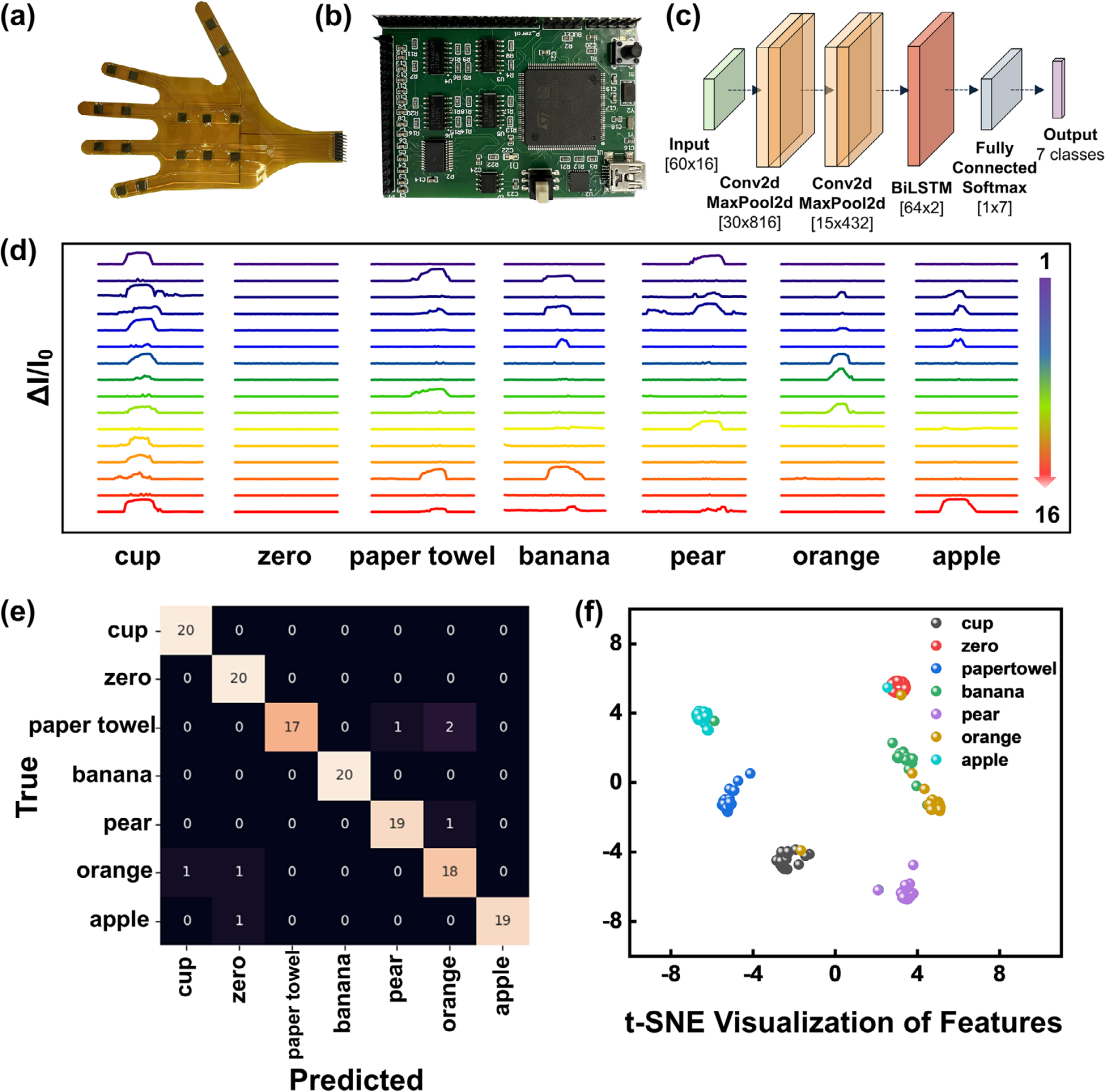
Object‐grasping recognition using a palm‐shaped sensor array based on the MSM structure. (a) Photograph of the 16‐channel palm‐shaped sensor array. (b) Photograph of the 16‐channel signal acquisition circuit board based on STM32F407. (c) Schematic diagram of the CNN–BiLSTM model architecture. (d) Example output signals of the palm‐shaped sensor array when grasping different objects. (e) Confusion matrix after 50 training epochs (test accuracy: 95%). (f) t‐SNE dimensionality‐reduction visualization showing the feature distribution of different object categories.

On the basis of the above applications, we fabricated a palm‐shaped 16‐channel sensor array to verify the integration and spatial resolution capability of the prepared sensor (Figure 4a). A volunteer wore the sensor array and grasped seven different types of objects—a cup, no object (denoted as “zero”), paper towel, a banana, a pear, an orange, and an apple. The current response signals were acquired through the A/D signal acquisition circuit (Figure 4b), and the 16‐channel signals elicited during each object‐grasping process were recorded. Following data collection, the CNN‐BiLSTM machine learning classification model (Figure 4c) underwent 50 training epochs. Figure 4d illustrates the waveform outputs of the palm‐shaped sensor array for each grasped object, with the corresponding channel‐sensor position mapping provided in Figure S11. The confusion matrix in Figure 4e confirms an overall recognition accuracy of 95%. Additionally, t‐SNE visualization of the extracted features (Figure 4f) shows that each object class forms a distinct cluster in the low‐dimensional space, which further corroborates the high recognition accuracy of the proposed sensor‐machine learning integrated system.

**FIGURE 5:**
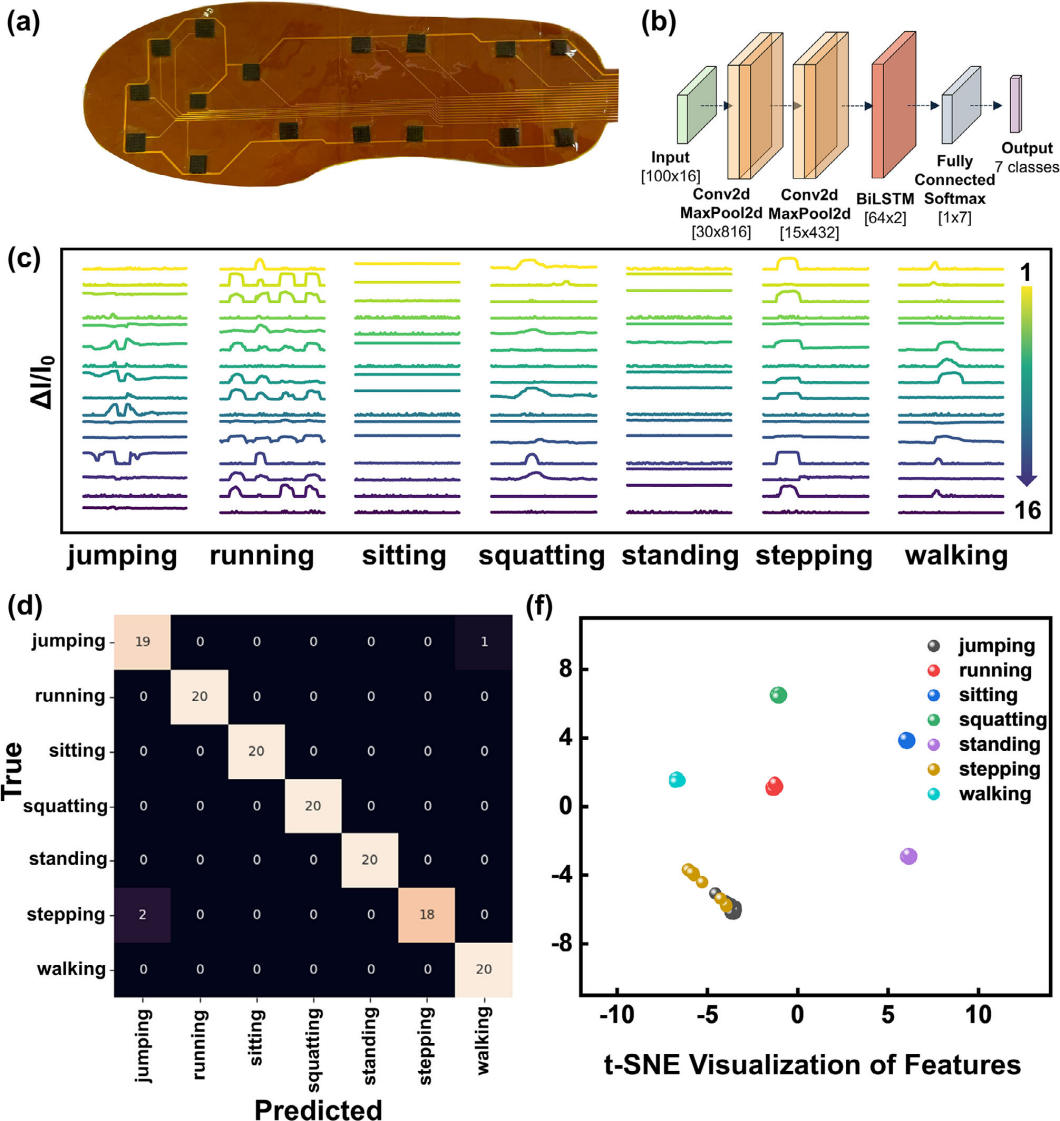
Motion‐pattern recognition using a sole‐shaped sensor array based on the MSM structure. (a) Photograph of the 16‐channel sole‐shaped sensor array. (b) Schematic diagram of the CNN–BiLSTM model architecture. (c) Output signals of the sole‐shaped sensor under different motion modes. (d) Confusion matrix after 50 training epochs (test accuracy: 97.9%). (e) t‐SNE dimensionality‐reduction visualization showing the feature distribution of different motion modes.

To further investigate the pressure‐resolution capability of the sensor array under large pressure ranges, we fabricated a sole‐shaped 16‐channel sensor array (Figure 5a) and integrated it into a sneaker. A volunteer wore the shoe equipped with this array and performed seven activities: jumping, running, sitting, squatting, standing, stepping, and walking. Using the CNN–BiLSTM machine‐learning classification model shown in Figure 5b, we trained the system for 50 epochs to distinguish different human gaits. The signals generated by the 16 channels during various motion states are shown in Figure 5c, and the mapping between the channel indices and the sole‐shaped array is provided in Figure S12. The confusion matrix in Figure 5d indicates that the system achieves a gait‐recognition accuracy of 97.9%, and the t‐SNE visualization in Figure 5e shows clearly separated clusters corresponding to different gait patterns. These results demonstrate that the prepared sensor array also exhibits exceptional resolution under high‐pressure conditions, which is of great significance for promoting the development of human–machine interaction and humanoid robotics.

## 4 Conclusion

In conclusion, we proposed an MXene‐based flexible piezoresistive sensor with a MSM structure, which incorporates cooperative primary microdome structure and secondary microdome structure together with a micro‐hemisphere array. Benefitting from the multi‐stage compression and stepwise contact feature of this architecture, the MSM‐based sensor realizes multi‐stage enhancement in pressure sensing, achieving an ultra‐wide detection range of up to 2500 kPa and a high sensitivity of 11.57 kPa^−^^1^ (111‐2500 kPa). The sensor exhibits response and recovery times of 82.6 ms and 41.4 ms, respectively, and undergoes no appreciable performance degradation after approximately 2900 cycles, thereby confirming superior dynamic performance and long‐term reliability. Furthermore, supported by deep‐learning algorithms, Morse code recognition, object grasping recognition, and human motion recognition are achievable with an overall recognition accuracy exceeding 95%. Overall, the proposed MSM structure offers a general structural strategy for advancing high‐performance flexible piezoresistive sensors, exhibiting great promise in wearable electronics, human–machine interaction, and robotic tactile perception.

## Conflicts of Interest

The authors declare no conflicts of interest.

## Supporting information




**Supporting file**: advs74927‐sup‐0001‐SuppMat.docx

## Data Availability

The data that support the findings of this study are available from the corresponding author upon reasonable request.
