# Supplementary material for: Flexible Pressure Sensor With Multi‐Stage Microdome Structure Enabling Ultra‐Wide Linear Range and High Sensitivity for Wearable Applications
Source: Adv Sci (Weinh). 2026 Mar 20;13(31):e74927. doi: 10.1002/advs.74927 (PMC13587947; doi:10.1002/advs.74927)
Supplement: Supplementary file 1 [file ADVS-13-e74927-s001.docx]

Supporting Information

**Flexible Pressure Sensor with Multi-stage Microdome Structure Enabling Ultra-Wide Linear Range and High Sensitivity for Wearable Applications**

De-Yu Kong^a#^, Kai Wang^a#^, Jia-Nan Ma^a,b*^, Bo-Ma^a^, Teng-Teng Gao^a^, Hai-Wang Zhang^a^, Hong-Tao Wang^a^, Qiang Li^a^, Hui Gao^a^, and Sheng-Bo Sang^a*^

a Shanxi Key Laboratory of Artificial Intelligence & Micro Nano Sensors, College of Integrated Circuits, Taiyuan University of Technology, Taiyuan 030024, China

b School of Materials Science and Engineering, Tianjin University, Tianjin 300350, China ‌

*Corresponding Author: Jia-Nan Ma ([majianan@tyut.edu.cn](mailto:majianan@tyut.edu.cn));

Sheng-Bo Sang (sunboa-sang@tyut.edu.cn).

\# These authors contributed equally to this work.

**Figure S1.** XPS full spectrum of MXene.

**Figure S2.** C1s spectra of MXene.

**Figure S3.** Three-dimensional characterizations of the fabrication process. (a) Three-dimensional optical microscopic scanning image of the positive mold through 3D printing. (b) Three-dimensional optical microscopic scanning image of the negative mold (Pullulan hydrogel). (c) Three-dimensional optical microscopic scanning image of the PDMS film with MSM structure before MXene coating.

**Figure S4.** Morphological and elemental analyses of the MSM structure without MXene coating. (a) SEM image of the MSM-structured PDMS film without MXene coating. (b) Enlarged SEM image of the primary microdome region corresponding to panel (a). (c) Ti elemental distribution map in the EDS mapping of the region shown in panel (b).

**Figure S5.** Equivalent circuit of the sensor.

**Figure S6.** Comparison of microdome structures with varying geometric configurations. (a) Single-stage microdome structure (height: 250 μm, no micro-hemisphere array). (b) Single-stage microdome structure (height: 500 μm, no micro-hemisphere array). (c) Two-stage microdome structure (no micro-hemisphere array). (d) MSM structure with a primary/secondary microdome height ratio of 1:0.3. (e) MSM structure with a primary/secondary microdome height ratio of 1:0.7. (f) MSM structure with a primary/secondary microdome height ratio of 1:0.9.

**Figure S7.** Electrical responses of sensors with different structural designs. (a) Output of sensors with different structures under various applied pressures. (b) Fitted results of the corresponding current-pressure responses.

**Table S1. Performance comparison of sensors with different structural designs.**

| **Structure** | **Pressure range (kPa)** | | **Sensitivity (kPa⁻¹)** | **R²** |
| --- | --- | --- | --- | --- |
| MSM structure (1:0.3) | 14–111 | | 0.62 | 0.927 |
|  | 111–1389 | | 5.45 | 0.966 |
| MSM structure (1:0.5) | | 14–111 | 5.02 | 0.961 |
|  |  | 111–1389 | 8.41 | 0.996 |
| MSM structure (1:0.7) | | 14–83 | 4.33 | 0.929 |
|  |  | 83–1389 | 7.65 | 0.995 |
| MSM structure (1:0.9) | | 14–69 | 1.32 | 0.952 |
|  |  | 69–1389 | 12.8 | 0.978 |
| Single-stage (500 μm) | | 14–1389 | 1.50 | 0.993 |
| Single-stage (250 μm) | | 14–1389 | 1.08 | 0.931 |
| Two-Stage | | 14–111 | 3.21 | 0.996 |
|  |  | 111–1389 | 3.71 | 0.995 |

**Figure S8.** Electrical responses of sensors with different MXene coating amounts. (a) Output of sensors with different MXene coating amounts under various applied pressures. (b) Fitted results of the corresponding current-pressure responses.

**Table S2. Performance comparison of sensors with different MXene coating amounts.**

| **MXene(mg/cm²)** | **Pressure range (kPa)** | **Sensitivity (kPa⁻¹)** | **R²** |
| --- | --- | --- | --- |
| **0.05** | 14–1389 | 0.026 | 0.633 |
| **0.1** | 14–111 | 0.68 | 0.981 |
|  | 111–1389 | 2.74 | 0.992 |
| **0.15** | 14–111 | 5.02 | 0.961 |
|  | 111–1389 | 8.41 | 0.996 |
| **0.2** | 14–111 | 5.57 | 0.972 |
|  | 111–1389 | 11.68 | 0.996 |
| **0.25** | 14–111 | 14.13 | 0.992 |
|  | 111–1389 | 4.76 | 0.822 |
| **0.5** | 14–83 | 5.23 | 0.922 |
|  | 83–1389 | 5.36 | 0.972 |
| **1** | 14–83 | 6.96 | 0.998 |
|  | 83–1389 | 2.31 | 0.928 |

**Figure S9**. Output of the sensor after storage for three days under different relative humidity conditions (Pressure: 700 kPa).

**Figure S10.** Long-term stability of the sensor.

**Figure S11.** The positions and corresponding channel assignments of the palm-shaped 16-channel sensor array (the numbers indicate the channel indices).

**Figure S12**. The positions and corresponding channel assignments of the sole-shaped 16-channel sensor array (the numbers indicate the channel indices).

**Dataset Description and Training Strategy for the Three Applications**

**Morse code dataset:** The input data are numerical arrays of size 400 × 1. The dataset contains 37 classes, with 100 samples per class. For each class, 80 samples were used for training and 20 samples for testing. The model was trained for 20 epochs with a learning rate of 0.001.

**Glove dataset:** The input data are numerical arrays of size 60 × 16. The dataset contains 7 classes, with 100 samples per class. For each class, 80 samples were used for training and 20 samples for testing. The model was trained for 50 epochs with a learning rate of 0.001.

**Planter dataset:** The input data are numerical arrays of size 100 × 16. This dataset also contains 7 classes, each with 100 samples. For each class, 80 samples were used for training and 20 samples for testing. The model was trained for 50 epochs with a learning rate of 0.001.
